# Supplementary material for: Identification of hippocampal area CA2 in hamster and vole brain
Source: bioRxiv. 2024 Feb 14:2024.02.12.579957. Preprint. [Version 1] doi: 10.1101/2024.02.12.579957 (PMC10888814; doi:10.1101/2024.02.12.579957)
Supplement: 1 [file NIHPP2024.02.12.579957V1-supplement-1.pdf]

bioRxiv preprint doi: https://doi.org/10.1101/2024.02.12.579957; this version posted February 14, 2024. The copyright holder for this preprint (which was not certified by peer review) is the author/funder, who has granted bioRxiv a license to display the preprint in perpetuity. It is made available under a CC-BY 4.0 International license.

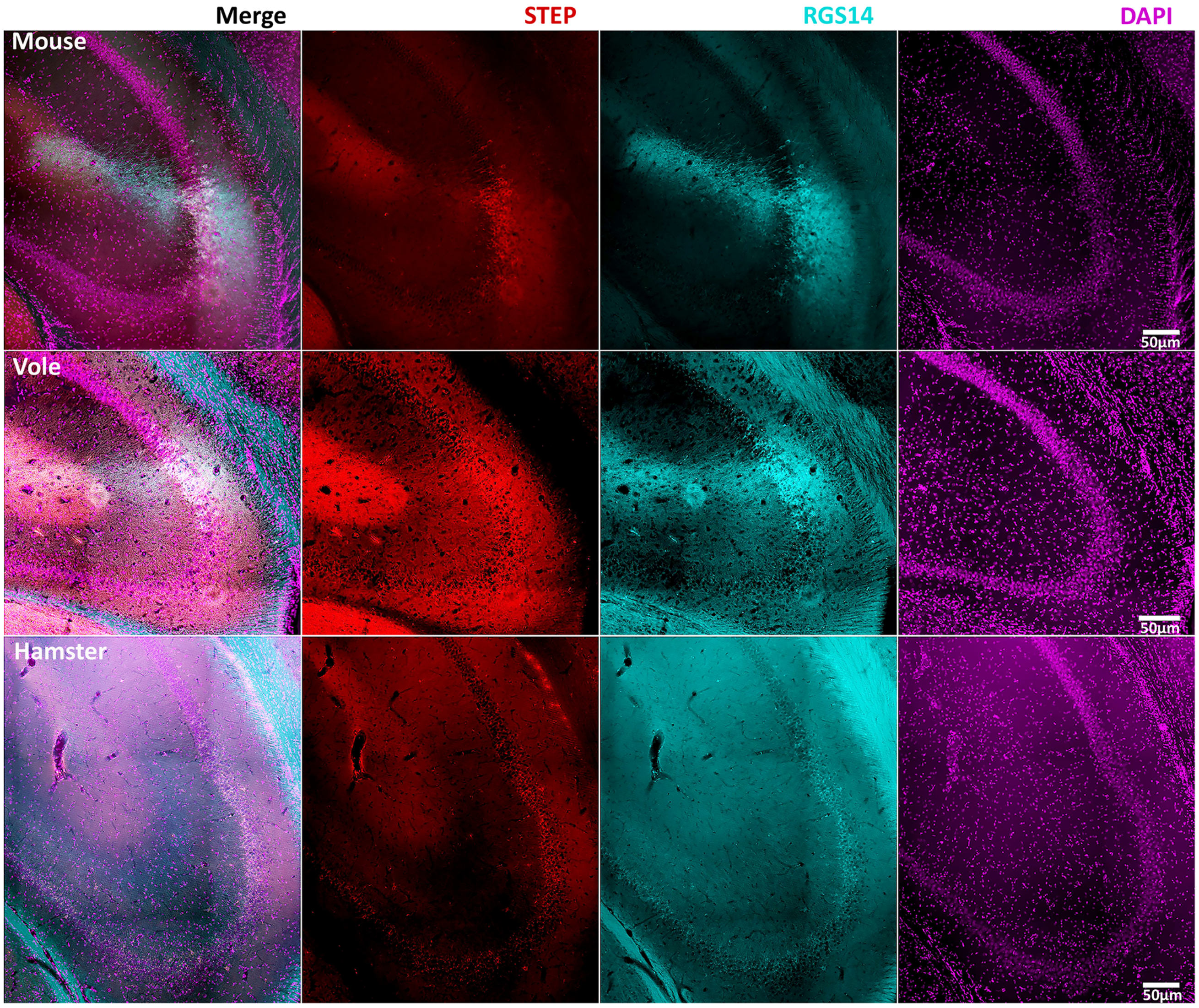

Supplemental Figure 1: RGS14 colocalizes with a putative area CA2 marker STEP.
Striatal-enriched protein tyrosine phosphatase (STEP) is present in dorsal CA2 in all three species and colocalizes with RGS14. STEP (red) is present in all three species, although it is faint in vole and hamster. RGS14 (cyan) demarcates area CA2, and DAPI (magenta) shows cellular density. Partial coronal hippocampal sections are shown.
